# Evidence-based, multilevel interventions for sustained behaviour change

**DOI:** 10.1016/S2214-109X(23)00372-8

**Published:** 2023-11

**Authors:** Bibhav Acharya, Sabitri Sapkota

**Affiliations:** Department of Psychiatry and Behavioral Sciences, University of California San Francisco, San Francisco, CA 94107, USA (BA); Sambhav (Possible), Bhim Plaza, Kathmandu, Nepal (SS)

The article by Rajshree Thapa and colleagues^1^ provides caution for potential harm in a 5-year follow-up of a hypertension-targeting lifestyle intervention delivered by community health workers in Nepal. Our research team is initiating a 600-person trial in Nepal^2^ on a behavioural intervention delivered by community health workers for comorbid noncommunicable diseases (NCDs; including hypertension and diabetes) and mental health conditions (depression and anxiety). The study is based on our 16 years of experience in behavioural change training of community health-care workers in Nepal.^3^ We would like to add our perspective to Thapa and colleagues’ excellent insights.^1^

First, most lifestyle interventions for NCDs are limited to advice giving and knowledge transfer from health-care workers to patient. Such interventions are common because it is easy to equip health-care workers with knowledge-based competencies (eg, listing the components of a healthy diet), which they can use to convey information to the patients (eg, “here are examples of vegetables you should eat”). However, there is well established evidence that individual-level educational approaches lead to transient or no behaviour change.^4^ Sustained behaviour change is reached by enhancing the patient’s internal motivation and creating a supportive environment that can turn motivation into healthy behaviours. Motivational interviewing has the most robust evidence for sustained behaviour change. This approach includes building a non-judgmental relationship with the patient, understanding the patient’s values and worldview, and facilitating the patient’s own desire to engage in healthy behaviours, such as improving diet or adhering to medications. ^3,5^ Unfortunately, community health workers (and most other health-care workers) rarely receive motivational interviewing training, despite the overwhelming evidence for this approach’s effectiveness. In the study by Thapa and colleagues^1^ the educational efforts and regular reminders likely caused participants’ transient behaviour change during the trial. However, the intervention group was possibly worse off than the control group at the 5-year point, because of absence of enhancement in internal motivation combined with self-perceived competence from knowledge acquisition, as noted by the study authors.^1^

The second contributor is related to the community health workers’ work environment. Even if community health workers’ interventions are effective during a study, they need to continue receiving support to ensure that their impact does not diminish after the study. In Nepal, community health workers are unpaid, excepting inconsistent cash incentives that are insufficient as the primary livelihood. Therefore, after the study, the workers often cannot continue to support patients who would have benefited from their services.

We commend the authors for this important follow-up study. Their findings provide caution for behavioural research globally. We will include two aspects in our study: using evidence-based behaviour change counselling (such as motivational interviewing) at the individual level and professionalising community health workers (ensuring salary and support) at the systems level.

## References

[1] Thapa R, Zengin A, Neupane D, Sustainability of a 12-month lifestyle intervention delivered by community health workers in reducing blood pressure in Nepal: 5-year follow-up of the COBIN open-label, cluster randomised trial. Lancet Glob Health 2023; 11: e1086–95. 37349035 10.1016/S2214-109X(23)00214-0

[2] Acharya B, Sapkota S. A type II hybrid implementation-effectiveness study of BECOME (behavioral community-based combined intervention for mental health and noncommunicable diseases) delivered by community health workers. National Institutes of Health Research Portfolio Online Reporting Tools (NIH report). 2023. https://reporter.nih.gov/search/Uv0PVqfeykeNdxb213tBWw/project-details/10658312 (accessed Aug 2, 2023).

[3] Rimal P, Khadka S, Bogati B, Cross-cultural adaptation of motivational interviewing for use in rural Nepal. BMC Psychol 2021; 9: 52. 33794990 10.1186/s40359-021-00557-y PMC8017825

[4] Rubak S, Sandbaek A, Lauritzen T, Christensen B. Motivational interviewing: a systematic review and meta-analysis. Br J Gen Pract 2005; 55: 305–12. 15826439 PMC1463134

[5] Miller WR, Rollnick S. Motivational interviewing: helping people change, 3rd edn. New York, NY: Guilford Press, 2013.

